# Model Studies on the Formation of the Solid Electrolyte Interphase: Reaction of Li with Ultrathin Adsorbed Ionic‐Liquid Films and Co_3_O_4_(111) Thin Films

**DOI:** 10.1002/cphc.202001033

**Published:** 2021-02-10

**Authors:** Katrin Forster‐Tonigold, Jihyun Kim, Joachim Bansmann, Axel Groß, Florian Buchner

**Affiliations:** ^1^ Helmholtz Institute Ulm Electrochemical Energy Storage (HIU) Helmholtzstraße 11 89081 Ulm Germany; ^2^ Institute of Surface Chemistry and Catalysis Ulm University Albert-Einstein-Allee 47 89081 Ulm Germany; ^3^ Karlsruhe Institute of Technology (KIT) P.O. Box 3640 76021 Karlsruhe Germany; ^4^ Institute of Theoretical Chemistry Ulm University Albert-Einstein-Allee 11 89081 Ulm Germany

**Keywords:** cobalt oxide, density functional theory, ionic liquids, solid electrolyte interphase, surface chemistry

## Abstract

In this work we aim towards the molecular understanding of the solid electrolyte interphase (SEI) formation at the electrode electrolyte interface (EEI). Herein, we investigated the interaction between the battery‐relevant ionic liquid (IL) 1‐butyl‐1‐methylpyrrolidinium bis(trifluoromethylsulfonyl)imide (BMP‐TFSI), Li and a Co_3_O_4_(111) thin film model anode grown on Ir(100) as a model study of the SEI formation in Li‐ion batteries (LIBs). We employed mostly X‐ray photoelectron spectroscopy (XPS) in combination with dispersion‐corrected density functional theory calculations (DFT‐D3). If the surface is pre‐covered by BMP‐TFSI species (model electrolyte), post‐deposition of Li (Li^+^ ion shuttle) reveals thermodynamically favorable TFSI decomposition products such as LiCN, Li_2_NSO_2_CF_3_, LiF, Li_2_S, Li_2_O_2_, Li_2_O, but also kinetic products like Li_2_NCH_3_C_4_H_9_ or LiNCH_3_C_4_H_9_ of BMP. Simultaneously, Li adsorption and/or lithiation of Co_3_O_4_(111) to Li_n_Co_3_O_4_ takes place due to insertion via step edges or defects; a partial transformation to CoO cannot be excluded. Formation of Co^0^ could not be observed in the experiment indicating that surface reaction products and inserted/adsorbed Li at the step edges may inhibit or slow down further Li diffusion into the bulk. This study provides detailed insights of the SEI formation at the EEI, which might be crucial for the improvement of future batteries.

## Graphical abstract

**The formation of the solid electrolyte interphase** at the electrode‐electrolyte interface is studied by X‐ray photoelectron spectroscopy and dispersion‐corrected density functional theory calculations. The interaction between an adsorbed ionic liquid, Li, and a Co_3_O_4_(111) thin‐film model anode reveals bis(trifluoromethylsulfonyl)imide and 1‐butyl‐1‐methylpyrrolidinium decomposition and lithiation of the cobalt oxide.

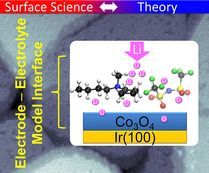

## 1 Introduction

In Li‐ion batteries (LIBs)[^1^, ^2^] the storage (or extraction) of Li in the anode conventionally occurs either via (de‐)intercalation or (de‐)insertion. A disadvantage of the traditional anode materials that make use of such storage mechanisms is their limited Li storage capacity, which is restricted by the available host sites. An alternative is the application of conversion materials like CoO and Co_3_O_4_. Transition‐metal oxides such as cobalt oxides are of significant interest as battery conversion anodes in LIBs[^3^, ^4^, ^5^, ^6^, ^7^, ^8^] in particular because they offer a remarkable Li storage capacity of up to around 900 mAh g^−1^,^[8]^ which is significantly higher than the capacity of standard electrodes.

However, a well‐known issue also occurring in conversion materials during battery operation is degradation,^[9]^ which is most likely related to massive structural changes (volume expansion) during phase transformation upon (dis‐)charging and breakdown of the solid electrolyte interphase (SEI). Yet, this issue is still unresolved. In general, for the storage/extraction of Li using Co_3_O_4_, it is basically unclear, whether Co_3_O_4_ irreversibly transforms into CoO and Li_2_O (phase transition) during the first cycle, and subsequently to Co^0^ and Li_2_O, or not. Lithiation during the first cycle could also start via filling the available insertion host sites and the subsequent reaction of lithiated Co_3_O_4_ to Co^0^ according to:

$$\mathrm{Li}{}_{n}\mathrm{Co}{}_{3}O{}_{4}+8\;\mathrm{Li}{}^{+}+8\;e{}^{-}\overset{\leftarrow }{\to }\;3\;\mathrm{Co}{}^{0}+4\;\mathrm{Li}{}_{2}O.$$

During the following cycles (de‐)lithiation/(de‐)conversion according to

$$\mathrm{CoO}+2\;\mathrm{Li}{}^{+}+2e{}^{-}\overset{\leftarrow }{\to }\;\mathrm{Co}{}^{0}+2\;\mathrm{Li}{}_{2}O$$

could occur.^[8]^ Furthermore, lithiation might proceed via the reversible reaction

$$\mathrm{Co}{}_{3}O{}_{4}+8\;\mathrm{Li}{}^{+}+8\;e{}^{-}\overset{\leftarrow }{\to }\;3\;\mathrm{Co}{}^{0}+4\;\mathrm{Li}{}_{2}O.$$

Hence, atomic or molecular insights into the processes of Li storage or extraction in/from the electrode of an operating battery would be very helpful.

Another crucial part for the function of a battery is the SEI that forms at the electrode electrolyte interface (EEI) due to decomposition of the electrolyte during cycling.[^1^, ^10^] It ensures a stable performance of LIBs, as the SEI is electronically non‐conducting and thus protects further electrolyte decomposition but still allows Li^+^ ion transport. Despite being essential, its composition and formation mechanism are not completely known yet. Thus, further atomistic/molecular understanding of the SEI formation together with a detailed knowledge of the chemical changes/transformation of Co_3_O_4_ is urgently demanded. Unfortunately, in real batteries (under operation conditions), these processes are not accessible by surface science techniques, which could provide such kind of information. In addition, at the cell level, deeper insights into the underlying mechanisms of the processes at the interface are hampered by the numerous components that constitute a standard electrolyte and the related large amount of possible processes occurring simultaneously. Therefore, in our model approach, we reduced the complexity significantly, and prepare well‐defined model electrodes like Co_3_O_4_ (111) thin film electrodes, under idealized ultrahigh vacuum (UHV) conditions, and explore the interaction of individual components of the battery electrolyte (e. g., ionic liquids, carbonates, lithium) using surface science techniques.[^11^, ^12^, ^13^, ^14^, ^15^, ^16^, ^17^, ^18^] In particular ionic liquids (ILs),[^19^, ^20^, ^21^, ^22^] that are organic salts with a melting point below 100 °C, got into the focus as promising solvents in battery electrolytes,[^23^, ^24^] as they are, for example, not flammable, such reducing the hazard of battery fires.^[25]^ Consequently, they represent an important class of compounds whose interactions with electrodes need to be studied.

Here we report results of a joint experimental and computational study employing mainly X‐ray photoelectron spectroscopy (XPS) in combination with density functional theory (DFT) calculations. Whereas we previously studied the interaction of an IL, Li and CoO(111),^[26]^ we now systematically expanded our investigations to Co_3_O_4_(111) studying its interactions with the same IL 1‐butyl‐1‐methylpyrrolidinium bis(trifluoromethylsulfonyl)imide (BMP‐TFSI) and the Li‐induced changes of the adsorbed IL and the substrate by post‐deposition of Li^0^, with the goal to mimic the initial stages of the chemical SEI formation at the EEI and the lithiation of an oxidic model anode in the absence of an applied potential (at the open circuit potential (OCP)).

However, before reporting on our results, we now shortly review previous studies related to this work. Concerning Co_3_O_4_(111), Luo and coworkers^[6]^ observed a phase transformation during the electrochemical (de‐)lithiation of Co_3_O_4_ nanocubes (∼5 nm) employing *in situ* transmission electron microscopy, revealing that smaller metallic Co nanoparticles embedded in a Li_2_O matrix form during lithiation (conversion). A computational study by Liu et al.^[27]^ reveals mechanistic details on the interaction of Co_3_O_4_(111) with Li, that is, first, Li is inserted at 16d octahedral sites (host sites) for small amounts of Li (Li_n_Co_3_O_4_, n≤1) and second, beyond insertion, Co_3_O_4_(111) is stepwise transformed into Li_2_O and CoO and finally to Co^0^ (conversion) for higher Li amounts. Another first principle calculation by Yao et al.^[28]^ identified a stable Li_n_Co_3_O_4_ (n≤3) insertion phase, which preserves a rigid oxygen framework during lithiation and which is proposed to have a constrained volume expansion and enhanced cycling stability at a capacity of ∼334 mAh g^−1^, before a conversion‐type reaction starts above 3 mol of Li per formular unit of Co_3_O_4_. In addition, several model studies were conducted on the interaction of BMP‐TFSI with different model electrode surfaces, and after Li exposure, e. g., on Cu(111),^[29]^ a copper foil,[^30^, ^31^] highly oriented pyrolytic graphite (HOPG),[^11^, ^13^, ^14^] TiO_2_(110),^[32]^ Si(111)^[33]^ and very recently on CoO(111).^[26]^ On CoO(111) stepwise post‐deposition of small amounts of metallic Li (<one monolayer equivalent (MLE)) to a pre‐adsorbed BMP‐TFSI adlayer results in the gradual decomposition of BMP‐TFSI into several decomposition products such as Li_2_S, LiF, LiC_x_H_y_N_z_ Li_2_NSO_2_CF_3_, SO_2_CF_3_. Only for higher Li amounts (>1MLE), relative to the amount of pre‐adsorbed BMP‐TFSI, electrolyte decomposition is followed by conversion of CoO(111) to Co^0^, demonstrating that the SEI layer (decomposition products) is permeable for Li.^[26]^

In a computational study employing ab initio molecular dynamics simulations to the adsorption of a TFSI‐based IL on Li Ando et al.^[34]^ showed that the S−C and C−F bond of TFSI break due to the interaction with the substrate and LiF is formed. Similar results are obtained by Yildirim et al.^[35]^ who found a cleavage of the C−S and N−S bond of TFSI due to a charge transfer from Li(100) to TFSI. In this study no bond breaking occurs for BMP.

Furthermore, studying the reduction of TFSI^−^ ions or of Li^+^−TFSI^−^ complexes by quantum chemistry methods Suo et al.^[36]^ also report the cleavage of the C−F and N−S bond of TFSI.

The present article is structured as followed: after a short summary of the experimental and computational methods that have been employed, we first validate the computational approach. Secondly we describe the Co_3_O_4_(111) thin films grown on Ir(100) (thickness ca. 9–12 nm), which were characterized by XPS and STM measurements in combination with DFT calculations. Then, we report our findings on (i) the interaction of Co_3_O_4_(111) with BMP‐TFSI, on (ii) the interaction of ultrathin BMP‐TFSI films with Li on a Co_3_O_4_(111) thin film model electrode and on (iii) the impact on the chemical state of the cobalt oxide substrate. We believe that such kind of molecular information on the surface chemistry going on at EEI is crucial for generating improved SEIs as an important step toward the development of better future batteries.

## Methods

### Experiment

The experiments were carried out in a commercial UHV system (SPECS) with a base pressure of 2×10^−10^ mbar. It consists of two chambers, one containing an Aarhus‐type STM/AFM system (SPECS Aarhus SPM150 with a Colibri sensor), the other one is equipped with an X‐ray source (SPECS XR50, Al‐K_α_ and Mg‐K_α_), a He lamp (SPECS UVS 300) and a hemispherical analyzer (SPECS, DLSEGD‐Phoibos‐Has3500) for XPS and UPS measurements.

Co_3_O_4_(111) thin films were prepared on a Ir(100) substrate (MaTecK, purity 99.99 %, surface roughness <0.01 μm, orientation accuracy <0.1°), following procedures described in literature.[^37^, ^38^, ^39^] Following Anic et al.,^[40]^ the Ir(100) sample was cleaned by Ar^+^ sputtering (1.7 keV, room temperature (r.t.)), annealing to 1370 K (4 min) and O_2_ adsorption at a temperature of 870 K in 5×10^−7^ mbar O_2_ (10 min). In a second step the sample was heated to 1370 K in UHV (6 min) and subsequently cooled down from 870 K to around r.t. in an O_2_ atmosphere (5×10^−7^ mbar), followed by a final flash annealing to 780 K in UHV, which (as verified by LEED measurements previously[^37^, ^38^, ^39^, ^40^]) results in a well‐ordered Ir (100)‐(2x1)O surface (note that in addition we observed Ir (100)‐(3×1)O). Afterwards, the Co_3_O_4_(111) thin films were grown on Ir(100)‐(2×1)O/Ir(100)‐(3×1)O by vapor deposition of metallic Co (Tectra twin pocket dual mini e^−^beam evaporator, equipped with 2 mm Co rod from Alfa Aesar 99,995 %) in a background atmosphere of O_2_ (oxygen 6.0, AIR LIQUIDE, ∼8×10^−6^ mbar) at r.t.. Subsequently, the films were annealed in an O_2_ atmosphere (∼8×10^−6^ mbar) at ∼520 K for 5 min and afterwards in UHV at around 570 K for 2 min. This preparation procedure resulted in clean Co_3_O_4_(111) films as determined by angle resolved XPS measurements (AR‐XPS) of the Co 2p, O 1s and Ir 4 f regions. A film thickness of around 9–12 nm was estimated from the evaporation rate of Co of around 4–6 Å/min together with the complete attenuation of the Ir 4 f signal at normal emission.

The IL (BMP‐TFSI, also referred to as C_4_C_1_Pyrr‐Tf_2_N,) was filled into a quartz crucible, which was mounted in a Knudsen effusion cell (Ventiotec, OVD‐3). Prior to its use, the IL was carefully degassed in UHV at around 400 K for 24 h to generate pure, water‐free IL. To generate IL adlayers on Co_3_O_4_(111), we evaporated the IL at a temperature of the IL source of 450 K. Under these conditions the deposition rate was ∼0.1 ML min^−1^, with 1 monolayer (ML) defined as a full layer at saturation coverage.

Lithium metal was deposited from an alkali getter source (SAES Getters), by resistively heating the source in line‐of‐sight of the sample. A highly oriented pyrolytic graphite test substrate cooled to 80 K (no intercalation) was used for calibration of the Li deposition rate.^[14]^ Deposition rates in MLE of approximately 0.04–0.05 MLE min^−1^ were calculated from the damping of the C 1s substrate) peak after successive vapor deposition of Li at temperatures where Li adsorbs on the surface. For the evaluation we assume that 1 MLE of Li has a thickness d of 2.48 Å, equivalent to the (110) interplanar distance in a body centered cubic lattice (the most stable configuration of a Li metal at r.t.). The layer thickness d was calculated by I_d_=I_0_ exp (−d/λ cos θ),(I_0_: C 1s peak intensity before Li deposition, I_d_: C 1s peak intensity after Li deposition, θ: emission angle with respect to the surface normal), with an electron inelastic mean free path (IMFP) λ for Li of 46 Å^[41]^ at the position of the graphitic carbon peak at kinetic energies of ∼1200 eV.

For the XPS measurements we used an Mg‐K_α_ X‐ray source (1253.6 eV), operated at a power of 250 W. XP spectra were recorded at a pass energy E_pass_ of 100 eV at normal and grazing emission (0° and 70° to the surface normal, respectively). For fitting the XP spectra we used the Igor Pro 8.03 software, which includes a simultaneous fit of background (Shirley+slope) and signal, assuming a pseudo‐Voigt type peak shape, which is a linear combination of a Gaussian and a Lorentzian function. The binding energy (BE) scale was calibrated by setting the position of the Ir 4f_7/2_ peak to 60.6 eV.^[42]^

### Computation

Periodic density functional theory calculations have been performed using the Vienna *ab initio* simulation package (VASP 5.4).[^43^, ^44^] The electron‐ion interaction was described by the projected augmented wave method.[^45^, ^46^] The electronic wave functions were expanded in a plane wave basis set up to a cutoff energy of 520 eV. The exchange and correlation energy was calculated within the generalized gradient approximation (GGA), employing the PBE functional^[47]^ and its revised version of Hammer and Nørskov (RPBE).^[48]^ Dispersion effects were included by means of the semi‐empirical correction scheme D3 of Grimme^[49]^ in connection with a damping function proposed by Chai and Head‐Gordon (“zero‐damping”).^[50]^ To account for on‐site Coulomb interactions a Hubbard like term (+U) was added in the way proposed by Dudarev.^[51]^ Note that the combination of RPBE‐D3+U can improve the description of polymorph stabilities of a metal oxide while keeping the U‐correction at low values and thus retaining the agreement of structural parameters with experiment.^[52]^ Thus we could use a relatively small value of U_eff_(=U−J) of 2.5 eV for the d‐electrons of Co in the Co_3_O_4_ surface, as described in section 3.1.

All geometry optimizations were carried out until all forces on atoms were less than 0.01 eV/Å. The electronic structure was converged within 10^−6^ eV. For the integration over the first Brillouin zone a Gaussian smearing of 0.05 eV has been used. Furthermore, calculations of bulk Co_3_O_4_, CoO and BMP‐TFSI employ 7×7×7, 5×11×5 and 2×1×1 k‐point meshs, respectively.

The Co^2+^ terminated Co_3_O_4_(111) surface is modelled by symmetric slabs of 11 atomic layers that are separated by a vacuum region of 20 Å. During geometry optimization the outer 3 atomic layers on both sides of the slab were allowed to relax while the inner 5 layers were kept fixed at their bulk positions. For surface calculations a 5×5×1 k‐point mesh has been employed. The simulation of the STM image was based on the approximation that the tunneling current is proportional to the local density of states (LDOS) close to the Fermi energy at the position of the tip according to the Tersoff‐Hamann scheme.^[53]^ Constant‐current images were simulated by an isosurface of the LDOS integrated between the Fermi energy and the sample bias (−2.3 eV). Calculations of adsorbed or intercalated Li atoms employ a (2×2) structure of the optimized Co_3_O_4_(111) surface slab and, due to the large size of the unit cell, only the gamma point for the integration over the first Brillouin zone. Using a 3×3×1 k‐point mesh changes the total energy by less than 0.5 meV/atom. Adsorbed and intercalated Li atoms are added to one side of the slab and dipole corrections are applied. The Li atom and the upper 3 (or 7) atomic layers of the surface slab were allowed to relax in case of Li adsorption (or insertion). Relaxing 7 instead of 3 atomic layers for structures in which Li is adsorbed leads to a change of less than 0.3 meV/atom in the total energy. The diffusion barrier of Li on Co_3_O_4_(111) has been extracted from the minimum energy path for diffusion processes between stable adsorption sites which were calculated by the climbing image nudged elastic band method.[^54^, ^55^]

## 2 Results and Discussion

### 2.1 Methodological Issues of the Computational Description of Co_3_O_4_ and BMP‐TFSI

To describe both the cobalt oxide surface and the ionic liquid at the same computational footing, we first looked at the performance of GGA functionals (augmented with corrections) with respect to bulk properties of Co_3_O_4_ and the ionic liquid BMP−TFSI.

**Figure 1:**
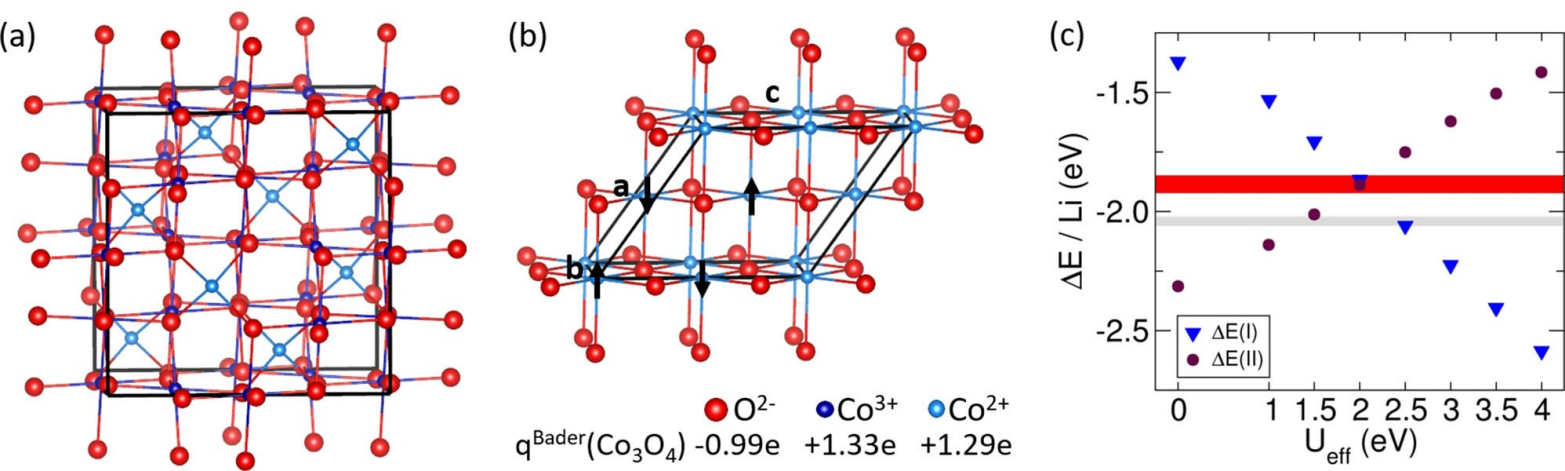
The spinel structure of Co_3_O_4_ (a) and the rock salt structure of CoO (b) are shown. The atomic charges of Co_3_O_4_ according to a Bader charge analysis are included in the Iegend. In (c) the reaction energy per Li atom ΔE/Li of the conversion reactions Co_3_O_4_+2Li→Li_2_O+3 CoO (I) and CoO+2Li→Co+Li_2_O (II) is shown as a function of the U_eff_ value. Horizontal lines or ranges denote experimentally determined values (grey: ΔE(I), red: ΔE(II)).

Co_3_O_4_ crystallizes in the spinel structure with Co^3+^ ions in octahedral sites and Co^2+^ ions in tetrahedral sites (Co^2+^Co^3+^_2_O_4_) that has been modeled by a cubic Co_24_O_32_ unit cell (see Figure 1a). It shows an antiferromagnetic arrangement of Co^2+^ ions and no magnet moment on Co^3+^ ions. Furthermore, the rock salt structure of CoO is studied using a unit cell structure that allows for the realization of a magnetic configuration according to type II antiferromagnetism (see Figure 1b). In order to determine a suitable U_eff_ value we compared the energies of the reactions

(I) $$\mathrm{Co}{}_{3}O{}_{4}+2\mathrm{Li}\to 3\mathrm{CoO}+\mathrm{Li}{}_{2}O$$

(II) $$\mathrm{CoO}+2\mathrm{Li}\to \mathrm{Co}+\mathrm{Li}{}_{2}O$$

to the respective experimental values (see Figure 1c).

The reaction energies per Li atom are calculated as

$$\Delta E\left(I\right)=(3E\left(\mathrm{CoO}\right)+E\left(\mathrm{Li}{}_{2}O\right)\text{-}E\left(\mathrm{Co}{}_{3}O{}_{4}\right)\text{-}2E\left(\mathrm{Li}\right))/2\Delta E\left(\mathrm{II}\right)=(E\left(\mathrm{Co}\right)+E\left(\mathrm{Li}{}_{2}O\right)\text{-}E\left(\mathrm{CoO}\right)\text{-}2E\left(\mathrm{Li}\right))/2$$

where E(X) denotes the energy obtained by RPBE‐D3+U for compound X ∈ {CoO, Li_2_O, Co_3_O_4_, Li, Co}. ΔE(I) and ΔE(II) are compared to reaction energies that have been calculated based on the experimentally deduced formation enthalpies of the compounds.[^56^, ^57^] For ΔE(I), the best agreement with the range of experimentally deduced reaction energies is found for U_eff_=2.5 eV, which leads to a deviation of about 0.1 eV for ΔE(II). On the other hand, ΔE(II) has the lowest deviation to experiment for U_eff_=2 eV, which leads to a deviation of 0.2 eV for ΔE(I).

Note, that we used the semiempirical dispersion correction (D3) on all compounds entering reactions I and II, though its usage for metallic systems is a matter of debate. We shortly discuss this issue and the results without dispersion corrections applied to metallic Li and Co in the Supporting Information (Figure S1). Furthermore, we also compared the lattice parameters of Co_3_O_4_ and CoO as well as the band gap of Co_3_O_4_ to their respective experimentally determined values (see Supporting Information, Figure S2, S3). Yet, the deviation of the lattice parameters from experiment varies only slightly for 1<U_eff_<4. Due to the fact that strongly different experimental values for the band gap of Co_3_O_4_ exist, a rigorous derivation of U_eff_ from the experimental band gap is not possible. Thus we mainly relied on the reaction energies to find a suitable value for U_eff_. Consequently, for all further calculations we chose a value of U_eff_=2.5 eV, which has been consistently derived using the same method for all compounds entering reactions (I) and (II). It yields a magnetic moment of 2.60 μ_B_ on the Co^2+^ atoms, comparable to magnetic moments reported previously.^[58]^ By a Bader charge analysis atomic charges of 1.33 e for Co^3+^, 1.29 e for Co^2+^ and −0.99 e for O^2−^ are obtained. Finally we note that by employing RPBE‐D3+U a smaller correction (U_eff_ in the range of 1.5–2.5 eV) is needed to obtain results comparable to experiment than by employing the PBE+U method, for which suitable values of U_eff_ in the range of 3.0‐3.5 eV are reported.[^58^, ^59^]

**Figure 2:**
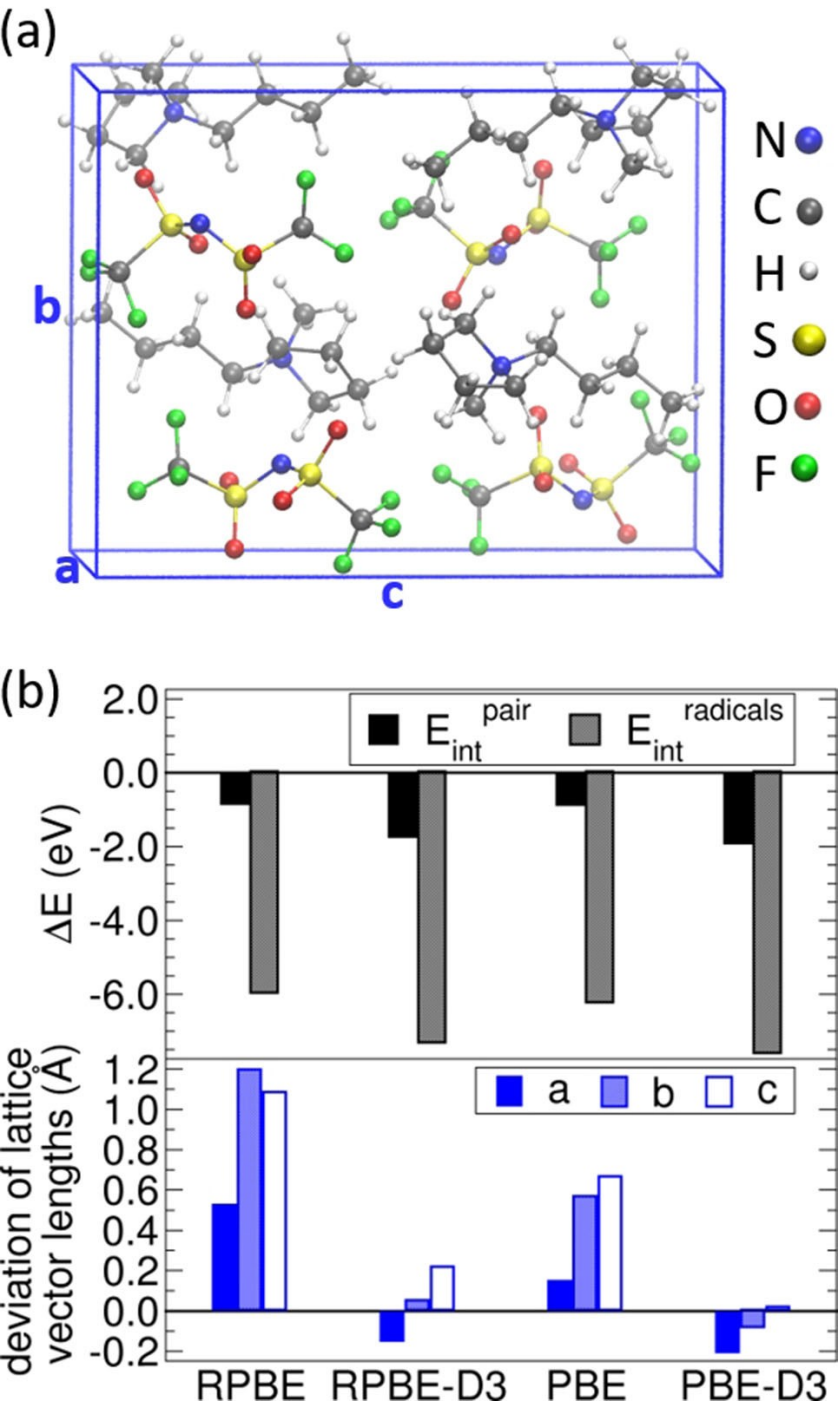
The orthorhombic crystal structure of BMP‐TFSI is shown in (a). The interaction energies of BMP‐TFSI in the crystal with respect to an ion pair (E_int_^pair^) and with respect to isolated BMP^0^ and TFSI^0^ radicals (E_int_^radicals^) as well as the deviation of the calculated lattice parameters from experiment (a=8.39 Å, b=13.01 Å, c=16.58 Å) are shown in (b).

Besides the necessity of a correction for the strongly correlated d‐electrons of Co, there is the need of a correction for dispersive interactions when it comes to ionic liquids.[^60^, ^61^] BMP‐TFSI crystallizes in an orthorhomic structure^[62]^ (Figure 2a). The unit cell of the BMP‐TFSI crystal consists of 4 BMP and 4 TFSI molecules that are arranged in a layered structure of alternating BMP^+^ and TFSI^−^ layers along lattice vector b. All TFSI ions show a *transoid* conformation, i. e. the CF_3_ groups are on opposite sides of the S−N−S plane. The lattice parameters of the BMP‐TFSI crystal significantly improve by adding dispersion corrections to the GGA functionals (Figure 2b, lower panel). In particular, the pure RPBE functional leads to a deviation of up to 9 % with respect to experiment, which reduces to less than 1.8 % if it is augmented by the semiempirical dispersion correction (RPBE‐D3). Furthermore, the impact of dispersion is clearly reflected in the interaction energies (Figure 2b, upper panel): by using pure GGA functionals the interaction energy of ion pairs within the crystal (E_int_^pair^=E(BMP‐TFSI‐crystal)/4‐E(BMP‐TFSI‐pair)) amounts to values of −0.85 eV (RPBE) or −0.88 eV (PBE).

These numbers are more than doubled when dispersion interactions are taken into account (E_int_^pair^ (RPBE‐D3)=−1.74 eV or E_int_^pair^ (PBE‐D3)=−1.93 eV). If the interaction energy is referred to the isolated radicals BMP^0^ and TFSI^0^ (E_int_^radicals^=E(BMP‐TFSI‐crystal)/4‐E(BMP^0^)‐E(TFSI^0^)), the differences in the interaction energies calculated by dispersion corrected and pure GGA‐functionals (which essentially corresponds to dispersion interactions) still make up 18 % of the interaction energies of the dispersion corrected calculations (E_int_^radicals^ (RPBE‐D3)=−7.30 eV, E_int_^radicals^ (PBE‐D3)=−7.59 eV). For both kinds of interaction energies we find that the PBE‐D3 yields a slightly stronger interaction than RPBE‐D3 (E_int_(PBE‐D3)‐E_int_(RPBE‐D3)=−0.2 to −0.3 eV). A similar effect has been seen for interactions within water, where comparison to experiment and quantum chemistry methods showed that the performance of dispersion augmented GGA functionals depends on the actual form of their exchange enhancement factor^[63]^ and, in particular, that RPBE‐D3 performs much better than PBE‐D3.[^64^, ^65^, ^66^, ^67^]

To summarize, we found the RPBE‐D3+U ansatz to be suitable to describe both cobalt oxides and the ionic liquid. As it is a numerically inexpensive method, it might further be used for calculations of extended surfaces and interfaces, where more sophisticated methods are not applicable.

### 2.2 Structure and Composition of the Co_3_O_4_(111) Films

**Figure 3:**
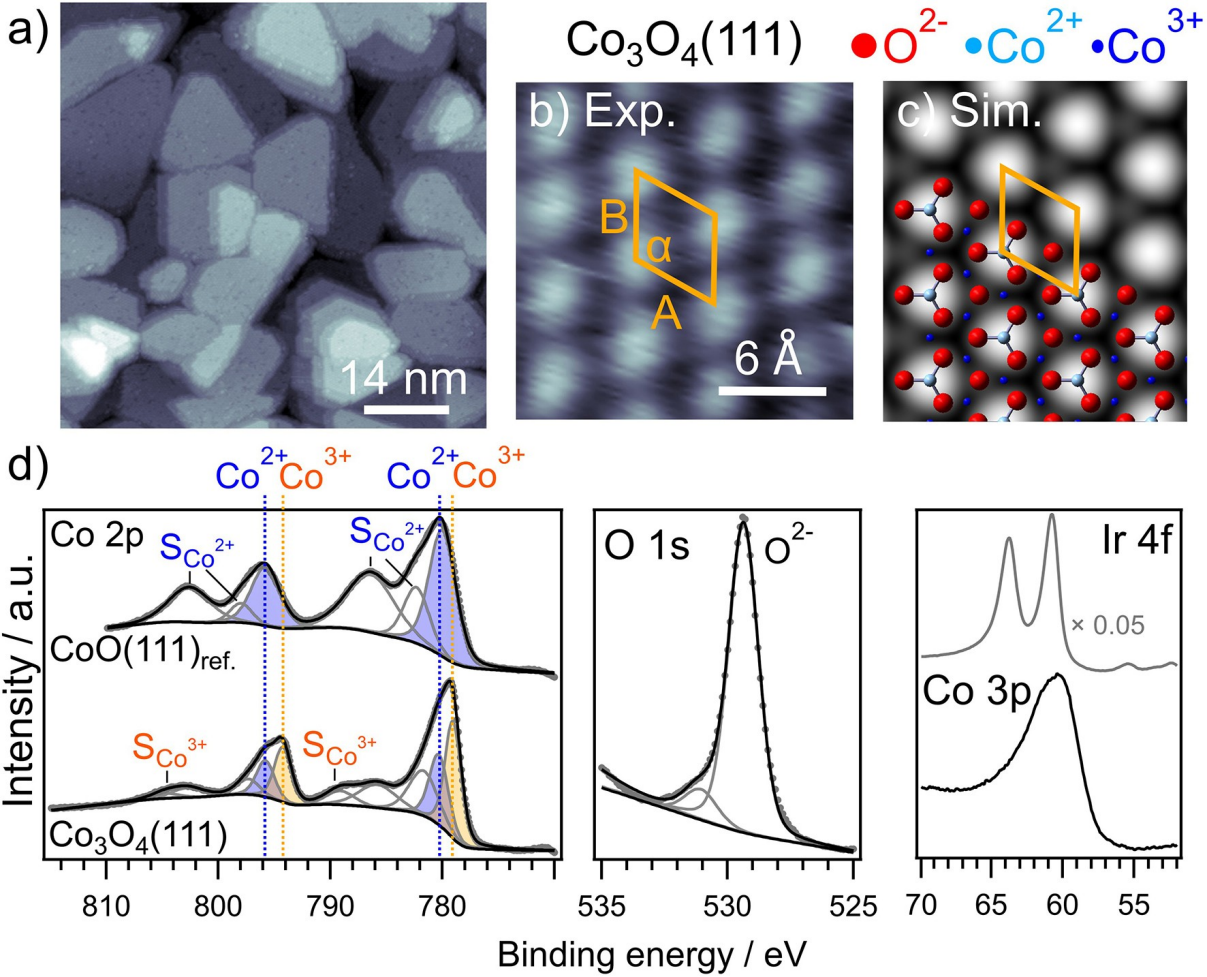
STM images (experiment: a‐b, simulation: c) and XPS measurements (d) recorded on a Co_3_O_4_(111) thin film on Ir(100)‐(2x1)O. The Ir 4 f level shows complete damping after preparation of Co_3_O_4_(111). The Co2p core level spectrum of CoO(111)_ref._ is also shown for comparison (d).

Experimentally, we first characterized Co_3_O_4_(111) thin films prepared on a Ir(100)‐(2×1)O/Ir(100)‐(3×1)O substrate (d_Co3O4_≈9–12 nm) by STM and XPS measurements (Figure 3) (details about the structure of oxygen on the Ir substrate can be found in refs. [68–69]). Our XPS measurements of Co 2p spectra were carried out at an emission angles of 0° with respect to the surface normal. In the following this is termed as normal emission. With an information depth (ID) of the Co 2p electrons of ∼6–9 nm an extended surface region is probed and thus it is also termed ‘bulk sensitive’. In case of adsorbate‐related spectra (F 1s, O 1s, N 1s, C 1s and S 2p peaks) measurements were done at an emission angle of 70° with respect to the surface normal. Further, this will be denoted as grazing emission. It is ‘surface sensitive’ as it probes the near surface region with an ID of about 2–3 nm.

In brief, the structure of these cobalt oxide films was tested by STM measurements prior to the adsorption experiments to verify planar, well‐defined Co_3_O_4_(111) model electrodes (Figure 3a,b). As displayed in the large‐scale STM image in Figure 3a, flat island structures could be resolved (similar STM images of Co_3_O_4_(111) thin films were previously shown in refs. [37–38,68]). The STM image in Figure 3b yields an atomic lattice with lattice vectors of |$\vec{a}$ |=|$\vec{b}$ |=0.57±0.03 nm and an angle of α=120±5°, in excellent agreement with results of previous reports.[^37^, ^38^] Based on I−V low energy electron diffraction (LEED) measurements in literature by Meyer et al.,^[38]^ the surface is terminated by Co^2+^ cations. Furthermore, dispersion corrected PBE+U calculations revealed such an oxygen rich surface termination with exposed Co at sites corresponding to tetrahedral bulk sites to be the most stable surface termination for an oxygen pressure larger than 10^−9^ bar at a temperature of 873 K.^[58]^ Thus, this termination of the Co_3_O_4_(111) surface has been used in our DFT calculations. The calculated lattice constant of Co_3_O_4_ using RPBE‐D3+U (U=2.5 eV) translates to a lattice vector length of 0.58 nm of the hexagonal surface unit cell in excellent agreement with the experiment. The simulation of the STM image of this surface (Figure 3c) indicates that the terminating Co atoms (corresponding to Co^2+^ atoms in the bulk, light blue ions in the model) are visible in the STM images.

The normal emission (0° with respect to the surface normal) Co 2p spectrum of the thin spinel Co_3_O_4_(111) film in Figure 3d reveals a fingerprint for Co_3_O_4_(111), with two asymmetric main peaks (doublet), which are related to Co^2+^ and Co^3+^ states in the Co 2p_1/2_ and Co2p_3/2_ regions, respectively, and a satellite structure for each of these peaks (cf. Biesinger et al.^[70]^). Deconvolution of the Co 2p spectrum (Figure 3d) by peak fitting (constraining the spin‐orbit split induced peak area ratio between Co 2p_1/2_ and Co2p_3/2_ to 1 : 2) leads to the following result: the doublet at 780.4 (Co 2p_3/2_) and 796.0 eV (Co 2p_1/2_) (filled blue), is assigned to Co^2+^ states together with two satellites for each peak (S_Co2+_) at ∼782, 786 and at 797, 802 eV, respectively.[^68^, ^70^] In addition, the Co 2p spectrum shows a doublet at 779.1 (Co 2p_3/2_) and 794.4 eV (Co 2p_1/2_) (filled orange), which is assigned to Co^3+^ states, together with one satellite for each peak (S_Co3+_) at ∼789 and 805 eV, respectively.^[70]^ The ratio of the peak intensities of the Co^3+^ : Co^2+^ peaks is ∼2 : 1, within the limits of accuracy, in good agreement with the nominal concentrations of these species in Co^2+^Co_2_^3+^O_4_. The O 1s region (Figure 3d) exhibits a main peak at 529.4 eV, which is due to lattice O^2−^ anions, and a low‐intensity shoulder at the high binding energy (BE) side (O_OH_), which we tentatively assign to adsorbed hydroxide. The ratio of the normalized peak intensities of the O^2−^ state and of the Co 2p region is ∼1.3, considering the atomic sensitivity factors of the O 1s (0.63) and Co 2p (4.5) signals, which matches with the expected nominal ratio. The normal emission Ir 4 f spectra (Figure 3d) recorded on the pristine Ir (grey solid line), show the Ir 4f_5/2_ and Ir 4f_7/2_ substrate peaks at 63.8 and 60.8 eV, respectively (multiplied by a factor of 0.05). After deposition of the Co_3_O_4_(111) film on Ir(100)‐(2×1)O (black solid line), the Ir 4 f features completely disappeared (for slightly shorter deposition times low intensity Ir peaks remained), which allows for the estimation of the film thickness of d ∼9–12 nm (see experimental part). As we will discuss changes of the chemical state of Co_3_O_4_ upon reaction with Li later in this work, we also display the Co 2p spectrum of a pristine CoO(111) thin film model electrode as a reference spectrum (cf. ref. [26,70–71]) (Figure 3d, top of the panel). The Co 2p spectrum shows a Co^2+^ doublet at 780.3 (Co 2p_3/2_) and 796.0 eV (Co 2p_1/2_) (filled blue), respectively, together with a pronounced satellite structure for each peak.^[70]^

### 2.3 Interaction of Co_3_O_4_(111) with BMP‐TFSI and Li

**Figure 4:**
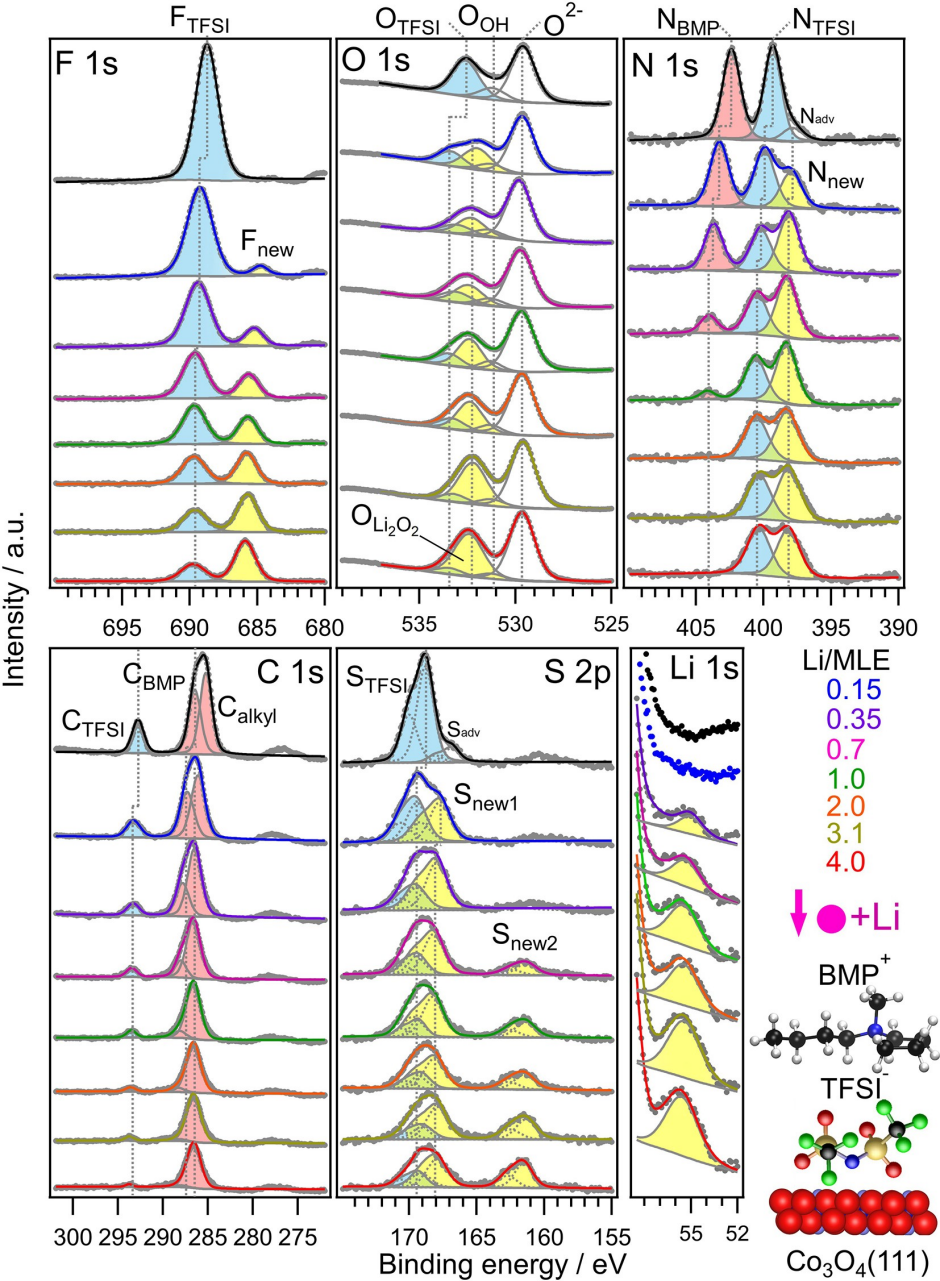
F 1s, O 1s, N 1s, C 1s, S 2p and Li 1s core level spectra of an adsorbed BMP‐TFSI adlayer (1.5–2 ML) (top of each panel) and upon stepwise post‐deposition of Li. A molecular presentation of BMP‐TFSI is inserted (fluorine: green, oxygen: red, nitrogen: blue, carbon: black, sulphur: gold).

Next, we vapor deposited an ultrathin film of BMP‐TFSI (1.5–2 ML) on Co_3_O_4_(111) at r.t. (Figures 4, top of each panel). In the resulting XP spectra (grazing emission, 70° with respect to the surface normal), BMP‐ and TFSI‐related peaks as shown in Table 1 appear.

**Table 1:** XPS peaks after vapor deposition of ∼1.5–2 ML of BMP‐TFSI (C_11_H_20_N_2_F_6_S_2_O_4_) on Co_3_O_4_(111).

Name		Group	BE [eV]	Nominal amount
F 1s	F_TFSI_	−CF_3_	688.8	6
O 1s	O_TFSI_	−SO_2_	532.5	4
N 1s	N_TFSI_	−S−N−S−	399.2	1
N 1s	N_BMP_	−C−N−	402.4	1
C 1s	C_TFSI_	S−CF_3_	292.9	2
C 1s	C_BMP_	−C−N−	286.3	4
C 1s	C_alkyl_	−C−C−	285.1	5
S 2p3/2	S_TFSI_	−N−SO_2_	168.9	2

The F_TFSI_, O_TFSI_, N_TFSI_, N_BMP_, C_TFSI_, C_BMP_, C_alkyl_ and S_TFSI_ peaks are due to molecularly adsorbed BMP‐TFSI species (cf. refs. [12,14,16]). A molecular representation of BMP‐TFSI is inserted in Figure 4 (bottom, right).

In the following, to mimic the Li^+^ ion transfer from the electrolytes into the electrode, Li^0^ (∼0.15–4 MLE) was successively post‐deposited on a Co_3_O_4_(111) film surface pre‐covered by a BMP‐TFSI adlayer (∼1.5–2 ML). XP core level spectra of the F 1 s, O 1s, N 1s, C 1s, S 2p and Li 1s spectral ranges are shown in Figure 4. We note that beside the BMP‐TFSI related peaks we also observed additional low‐intensity features (N_adv,_ S_adv_) which we tentatively assign to small amounts of BMP‐TFSI decomposition products caused by defects in the oxide film, most likely due to incomplete oxidation of the Co species. Similar peaks due to IL decomposition were previously also observed upon deposition of BMP‐TFSI on CoO(111).^[26]^ We also note that we observed moderate BE up‐shifts of all BMP‐TFSI related peaks (indicated by vertical dashed lines in Figure 4) during post‐deposition of Li^0^, which was very similarly observed in our previous publications upon Li exposure of carbonate and IL covered HOPG surfaces and which was assigned to vacuum level pinning.[^11^, ^17^]

To begin with, in the F 1s range in Figure 4 it is clearly visible that the F_TFSI_ peak gradually decreases in intensity upon increasing the Li dose from around 0.15–4.0 MLE of Li. At the same time a new peak arises at the low BE side (filled yellow), which grows with increasing Li exposures. The new peak (BE of 686 eV after the last Li dose) is assigned to LiF due to the cleavage of the −CF_3_ bond of the TFSI anion upon reaction with Li. After post‐deposition of 0.15 MLE of Li the F_TFSI_ peak decreased by 28 % and by 87 % after the last Li deposition step. The total peak area decreases more moderately by 24 % after deposition of 0.15 MLE of Li and by 60 % after the last deposition step, which can be explained by the transformation of molecularly adsorbed IL species into a decomposition product and desorption processes, i. e., 52 % desorption and 35 % decomposition, however, note that a decrease of the peak area could maybe in parts also be related to damping effects induced by adsorbed Li species.

We also show the Li 1s spectrum, which displays stepwise increasing peak areas for increasing amounts of deposited Li at a BE of ∼56.5 eV. For metallic Li we expected BEs of ∼55.5 eV.^[14]^ However, Li^0^ could, at least in parts, contribute to the Li 1s spectrum, nevertheless, the main contribution is most likely related to reaction products upon interaction of BMP‐TFSI with Li. Unfortunately, in the Li 1s spectrum, different Li‐containing species cannot be resolved because of the relatively poor intensity of this peak, due to the low photoionization cross‐section of the Li 1s core level.

In the O 1s range the O_TFSI_ peak also decreases in intensity as well, when the Li dose is stepwise increased. Most significant is the emergence of a new peak (indicated in yellow) at around 532.5 eV, which we assign to the formation of Li_2_O_2_. Qiu et al.^[72]^ observed a Li_2_O_2_ peak (532.5 eV) after deposition of O_2_ on a Li film. The new peak in our experiment could be either formed due to a break of the −SO_2_ bond of the TFSI anion upon reaction with Li, upon reaction with Co_3_O_4_(111) or after reaction of unreacted Li with residual species in the UHV chamber. Here quantitative information on the decrease of the O_TFSI_ is hardly possible due to the close proximity of the O_TFSI_ and the Li_2_O_2_ peaks. Note that we cannot probe Li_2_O due to the overlap of this peak with the O^2−^ peak (∼529.8 eV) of Co_3_O_4_(111) (cf. ref. [26]).

In the N 1s range the N_BMP_ and N_TFSI_ peaks simultaneously decrease in intensity up to 0.35 MLE of Li (−46 %). Now, a new peak (N_new_) evolves at ∼398 eV at the equal cost of both N_BMP_ and N_TFSI_ peaks. Though a rigorous assignment of this new peak is difficult, we tentatively propose that LiCN might be formed. Related compounds such as the lithium tetracyanoquinodimethane charge‐transfer complex (Li−TCQN) shows a BE of 398.6 eV^[73]^ and KCN of 398 eV.^[74]^ After post‐deposition of ≥0.7 MLE of Li, preferentially the N_BMP_ peak stepwise decreases in intensity and is almost completely absent after the last Li dose. Interestingly, at this point, the N_TFSI_ peak does not decrease any further, even if for all other TFSI‐related peaks, we monitored a continuous decrease upon increasing the amount of Li (see description of F_TFSI_ and O_TFSI_ above and C_TFSI_ and S_TFSI_ below in the text). Hence, we assume that at least part of the N_TFSI_ peak (cyan) is related to a decomposition product either of TFSI or of BMP. A possible first TFSI decomposition product might be Li_x_NSO_2_CF_3_, which could have been formed by dissociation of the S−N bond and subsequent bonding with Li. Such a species would be expected to exhibit a rather similar BE with the former N_TFSI_ peak and hence cannot be resolved in our experiment.

A possible BMP decomposition product would be Li_x_NC_y_H_z_, which requires C−N bond breaking. Studying reduction reactions of BMP by DFT calculations, Haskins et al.^[75]^ found that all C−N bond dissociations are favorable. Taking entropic contributions into account, the most favorable reaction is the formation of methylpyrrolidinium and a butyl radical. Note, that structurally related amines (R_x_NH_y_) show binding energies in the N 1s range of about 401.7 eV (tertiary amines, R_3_N^[76]^), 400.1–400.3 eV (secondary amines, R_2_NH[^76^, ^77^]) or 398.9–399.2 eV (primary amines, RNH_2_[^76^, ^78^, ^79^]). Thus the N 1s BE of decomposition products of BMP might be in the same range as the N 1s BE of TFSI. Considering also the F 1s and C 1s ranges (see description above and below) it is more likely that the former N_TFSI_ peak is now mainly due to decomposition products of BMP (Li_x_NC_y_H_z_). The total peak area decreases again moderately by 12 % after deposition of 0.15 MLE of Li and by 37 % after the last deposition step.

In the C 1s range it is clearly visible that also the anion‐related C_TFSI_ peak (−CF_3_ bond) gradually decreases in intensity upon increasing the Li dose (0.15–4.0 MLE of Li). After post‐deposition of 0.15 MLE of Li the C_TFSI_ peak decreased by 42 % and almost completely after the last Li dose. Going to the C_BMP_ and C_alkyl_ peaks, a quantitative analysis during Li exposure is hardly possible because of the broad feature between 285 and 290 eV induced by these species, which can principally be fitted by two peaks of varying intensity. Hence, we correlated the intensity changes of the C_BMP_ peak with that of the N_BMP_ peak, while the C_alkyl_ peak was free floating. After the last Li dose only a small amount of C_alkyl_ remains. The total peak area decreases by 11 % after deposition of 0.15 MLE of Li and by 69 % after the last deposition step.

Moving to the S 2p range, the S_TFSI_ doublet gradually decreases in intensity upon increasing the Li dose, while two new peaks (S_new1_ and S_new2_) emerge. After the first Li dose (0.15 MLE), the S_new1_ doublet arises with a BE of the S 2p_3/2_ peak at ∼168 eV, the second new doublet (S_new2_) sets in at 161.7 eV after deposition of 0.7 MLE of Li. After post‐deposition of 0.15 MLE of Li the S_TFSI_ peak decreased by roughly 20 % and by ∼70 % after the last Li deposition step (we note that the relatively broad feature between 166 and 172 eV allows peak deconvolution by various different intensity ratios of the S_TFSI_ and the S_new1_ peak, hence the evaluated values have to be treated with caution). The total peak area did almost not change after deposition of 0.15 MLE. The total intensity loss after the last Li exposure is ∼20 %.

Overall, during stepwise postdeposition of approximately 0.15–4.0 MLE of Li all TFSI‐related peaks gradually decrease in intensity, reflecting the decomposition of the anions. The cation‐related N_BMP_ peak also reveals a pronounced intensity loss. The loss of intensity of the anion‐ and cation‐related peaks in the XP spectra is accompanied by the formation of new peaks (filled yellow), reflecting the formation of various decomposition products. Based on their BEs, possible products are LiCN (N 1s, 398.5 eV), LiF (F 1s, 685.7 eV), Li_2_O_2_ (O 1s, 532.3 eV) and Li_2_S (S 2p_3/2_, 161.8 eV) as well as other Li bonded fragments of the anion such as Li_x_NSO_2_CF_3_ or the cation such as Li_x_NC_y_H_z_. The formation of these Li‐bonded fragments is associated with the initial stages of the chemical SEI formation. In general, in our previous experiments we observed similar new peaks (decomposition products) on different model electrode surfaces such as HOPG,[^11^, ^13^, ^14^] or CoO^[26]^ during postdeposition of Li on pre‐adsorbed BMP‐TFSI adlayers, demonstrating that the IL decomposition takes place independent of the substrates. In addition to the transformation of BMP‐TFSI into decomposition products on the surface, the interaction with Li must also result in the formation of volatile species, which desorb upon formation as evident from the decreasing total peak areas, however damping effects induced by adsorbed Li species might also partially contribute to the decreased peak area.

**Figure 5:**
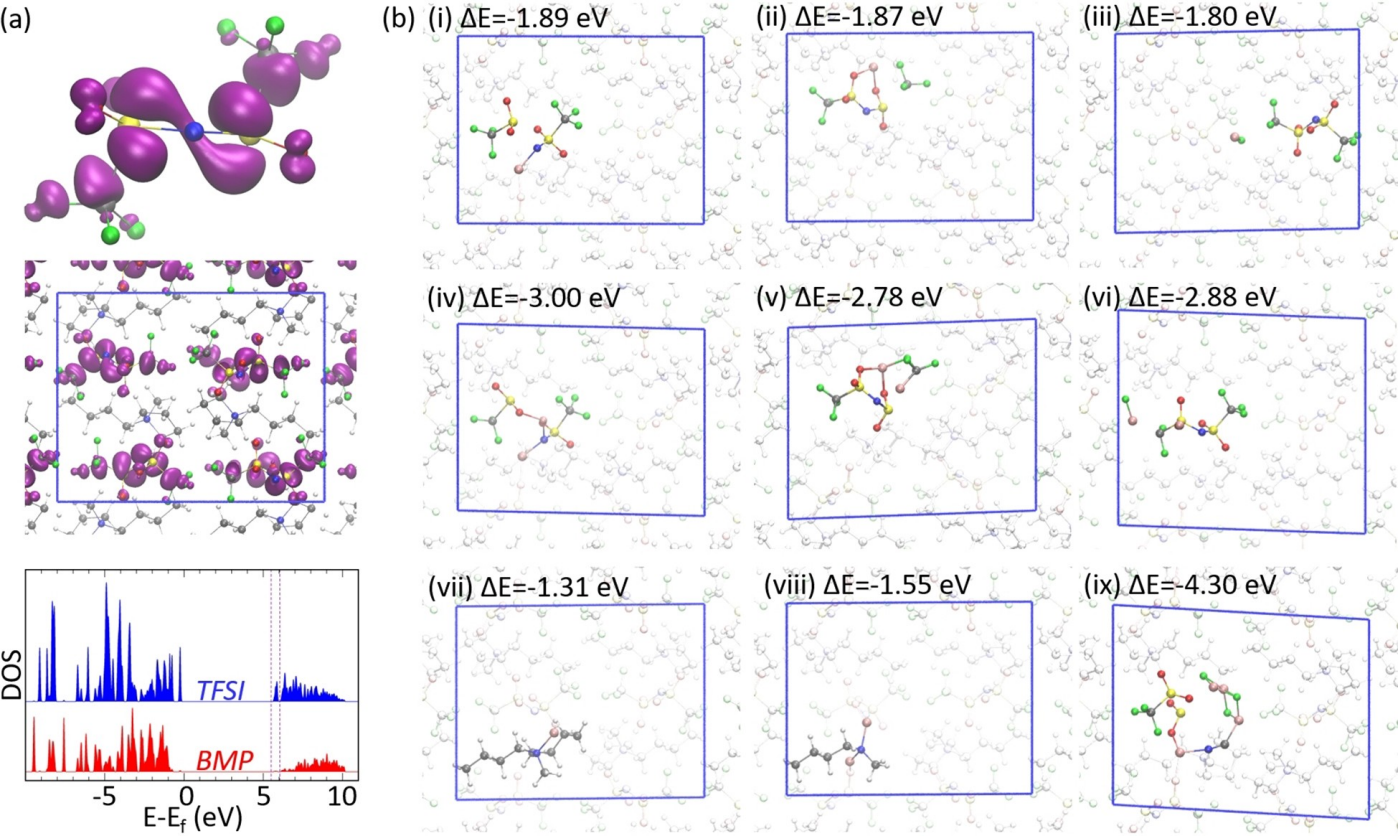
(a) The electronic density of states (DOS) of the BMP‐TFSI crystal projected onto the atoms of BMP and TFSI (bottom), along with an isosurface (isosurface value=0.02 e) of the partial charge density in the energy range of the lowest unoccupied states (5.50–6.04 eV) within the whole unit cell (middle) and enlarged at TFSI (top). (b) Different decomposition structures of TFSI (i–vi, ix) and BMP (vii,viii) within the BMP‐TFSI crystal due to the reaction with one (i–iii,vii), two (iv–vi,viii) or four (ix) Li‐atoms inserted into the pure IL crystal structure. The reaction energy per Li atom is given with respect to the BMP‐TFSI crystal and an isolated Li atom (ΔE=(E(decomposed structure)‐E(BMP‐TFSI‐crystal)‐E(Li‐atom))/x; x=(1,2,4)). Additionally, as far as the decomposition of BMP due to the reaction with two Li atoms is concerned, the formation of two ethylene molecules that desorb into the gas phase is assumed.

To shed some light on possible first reaction products of BMP‐TFSI and Li, we employed DFT‐D3 calculations of the BMP‐TFSI crystal structure in which we inserted up to four Li atoms. By looking first at the electronic properties of the neat BMP‐TFSI crystal (see Figure 5a), we find that the partial electronic density in the energy range of the lowest unoccupied molecular orbital shows only contributions of the TFSI anion. In detail, it is composed of contributions of the nonbonding S−N−S π orbital, as well as the antibonding C−F and S−C orbitals. Thus, structures with one or two Li atoms inserted in broken N−S, C−F and S−C bonds of the TFSI anion in the BMP‐TFSI crystal structure were used as input structures for DFT‐D3 geometry optimization calculations. For comparison, structures with decomposed BMP cations were also considered. Reaction energies per Li atom (ΔE) of the decomposition products (E(product)) have been calculated with respect to Li atoms in the gas phase (E(Li‐atom)) and the BMP‐TFSI crystal (E(BMP‐TFSI)):

$$\Delta E=\left(E\left(\mathrm{product}\right)\text{-}E\left(\mathrm{BMP}\text{-}\mathrm{TFSI}\right)\text{-}n{}_{\mathrm{Li}}E\left(\mathrm{Li}\text{-}\mathrm{atom}\right)\right)/n{}_{\mathrm{Li}}$$

where n_Li_ denotes the number of Li‐atoms. Results are shown in Figure 5b. According to our DFT‐D3 calculations, the decomposition of TFSI is more favorable than the reaction of BMP with Li. This is in agreement with previous computational studies of the reduction of BMP‐TFSI by Li^[35]^ and allows us to exclude a lower reactivity of TFSI compared to BMP regarding the reaction with Li, which at first sight might be deduced by looking at the evolution of the peaks in the N 1s BE range of the experimental XP spectra (Figure 4) with increasing Li dose: while the N_BMP_ peak gradually vanishes, the intensity of the N_TFSI_ peak seems to remain stable after a certain amount of Li is added (see description in the text above). In particular, among the various decomposition products that are studied, Li_2_NSO_2_CF_3_ (structure iv in Figure 5b) turns out to be the most stable initial product with a reaction energy per Li atom of −3.0 eV. Yet, regarding the reaction with only one Li atom, structures with dissociated S−C or C−F bonds are only about 0.02 or 0.09 eV less stable than the structure with the dissociated N−S bond. A further decomposition product of TFSI obtained by geometry optimizations of BMP‐TFSI with 4 Li atoms per unit cell is LiCN (see structure ix of Figure 5b). Its large reaction energy per Li atom of −4.30 eV hints at a remarkable thermodynamical stability. However, the reaction barrier of the N−S and C−S bond breaking and C−N bond formation needed to form LiCN has not been determined yet because of the complexity of the reaction mechanism. Decomposition products of BMP (structures vii and viii in Figure 5b) are much less stable with reaction energies of −1.31 eV and −1.55 eV whose absolute values are in the range or even smaller than the cohesive energy of bulk Li (E_coh_^RPBE^=1.54 eV, E_coh_^RPBE‐D3^=1.71 eV). Thus, thermodynamically, the formation of Li clusters is at least as favorable as the decomposition of BMP. However, due to a low barrier for the reaction of Li with BMP‐TFSI, which was already reported for the decomposition of BMP‐TFSI monolayers at graphite,^[11]^ kinetic products are likely to be formed as well. Thus, if BMP gets decomposed, the formation of Li_2_NCH_3_C_4_H_9_ due to the reaction of 2 Li atoms with BMP is probable. Along with the formation of Li_2_NCH_3_C_4_H_9_ two ethylene molecules desorbing into the gas phase are generated and Li_2_NCH_3_C_4_H_9_ retains the positive charge of BMP. The corresponding neutral moiety LiNCH_3_C_4_H_9_, which may form by releasing Li^+^, resembles in its structure and in the environment of N a secondary amine and thus might also have a similar N 1s core level binding energy. In order to see whether the computationally determined stable reaction products are compatible with experimental XPS results, a first estimate of the qualitative order of the N 1s core level BEs of the most stable decomposition products might be given by the atomic charges. Therefore, here we compare the number of electrons within R_wigs_=0.741 Å around N of BMP, TFSI and different decomposition products within the BMP‐TFSI crystal:

$$n{}_{e}\left(\mathrm{BMP}\right)=3.835<n{}_{e}\left(\mathrm{Li}{}_{2}\mathrm{NCH}{}_{3}C{}_{4}H{}_{9}\right)=3.837<n{}_{e}\left(\mathrm{Li}{}_{2}\mathrm{NSO}{}_{2}\mathrm{CF}{}_{3}\right)=3.866<n{}_{e}\left(\mathrm{TFSI}\right)=3.892<n{}_{e}\left(\mathrm{LiCN}\right)=3.899.$$

The trend tentatively suggests that among the compounds studied, BMP shows the highest BE of the N 1s electron, while LiCN exhibits the lowest one. According to that, we are inclined to assign the new peak at a BE of 398.5 eV to LiCN that forms due to a decomposition reaction of TFSI with Li. Besides, we propose that there is at least one other new peak at a BE presumably only slightly higher than the BE of the N_TFSI_ peak, which might be due to the decomposition products Li_2_NCH_3_C_4_H_9_ and/or Li_2_NSO_2_CF_3_. Note that a rigorous correlation of the atomic charges to the core level BE can be difficult, as final state effects might be crucial. Yet, a more detailed analysis of further decomposition products of BMP‐TFSI due to the reaction with Li and the corresponding core level shifts is beyond the scope of this article and will be discussed elsewhere.

**Figure 6:**
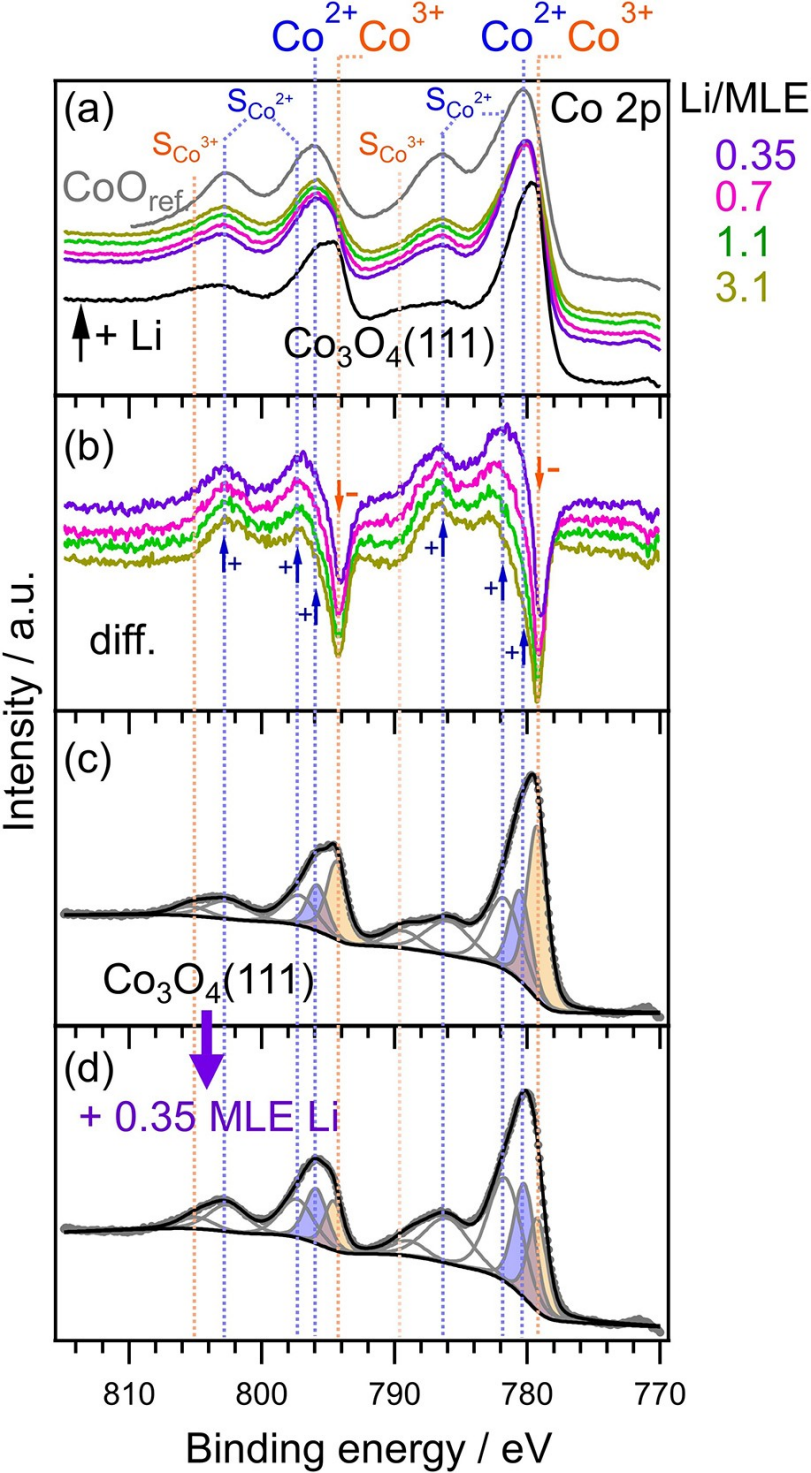
Co 2p core level spectra of an adsorbed BMP‐TFSI adlayer (1.5–2 ML) on Co_3_O_4_(111) (black) and upon stepwise post‐deposition of Li (a), Co 2p difference spectra between the spectrum of pristine Co_3_O_4_(111) and after each Li deposition step (b), peak deconvoluted Co 2p spectrum of pristine Co_3_O_4_(111) (c) and after post‐deposition of 0.35 MLE of Li (d).

In the XPS experiment, during the stepwise postdeposition of 0.15–4.0 MLE of Li on Co_3_O_4_(111) pre‐covered by a BMP‐TFSI adlayer (1.5–2 ML), we also monitored the Co 2p regime to test for changes of the chemical state of the thin film model electrode (Figure 6). Figure 6a displays the Co 2p range for pristine Co_3_O_4_(111) (black solid line, bottom of the panel) and after deposition of 0.35, 0.7, 1.1 and 3.1 MLE of Li. It is clearly visible that already after exposure of 0.35 MLE of Li (violet solid line) the two Co 2p maxima shift from around 779.7 and 794.1 eV to 780.2 and 796.0 eV, respectively. Simultaneously the two S_Co2+_ satellites significantly increase in intensity. For higher Li doses no significant changes occur. In addition, we show the corresponding XP difference of the XP spectra during Li deposition minus the spectrum of pristine Co_3_O_4_ (Figure 6b). They reveal peaks with positive amplitudes (intensity gain) after deposition of 0.35 MLE of Li (violet solid line) at the position of Co^2+^ states and their related satellites as well as peaks with negative amplitudes (intensity loss) at the BEs of Co^3+^. After post‐deposition of 0.7, 1.0 and 3.1 MLE of Li, the Co^3+^ loss is only slightly enhanced, and only a marginal decrease of the two S_Co2+_ satellites becomes visible, which is maybe due to damping effects induced by adsorbed Li species. The BE shift from Co^3+^ to Co^2+^, that is, an increasing amount of Co^2+^ relative to Co^3+^ can be ascribed to charge transfer from Li^0^ to Co^3+^ due to insertion or/and adsorption. Indeed, Yao et al.^[28]^ calculated decreasing atomic charges of Co with increasing Li insertion in Co_3_O_4_. Their proposed mechanism includes Li insertion into available host sites, leading to Li_n_Co_3_O_4_ up to n=3 and Co^3+^ to Co^2+^ reduction. For n=3 they found a mosaic structure with local Li_2_O and CoO character, for larger Li content the local transformation to Li_2_O and CoO and finally to Co^0^.

Peak deconvolution of pristine Co_3_O_4_(111) before (Figure 6c) and after deposition of 0.35 Li (Figure 6d) reveals that the initial Co^3+^ : Co^2+^ ratio of 2 : 1 for the pristine cobalt oxide significantly changes. For the peak deconvolution we constrained the ratio between the Co^2+^ peaks and their related S_Co2+_ satellites (∼786 and 802 eV, respectively) to ∼1.2, that is, the ratio used for deconvolution of both pristine CoO and Co_3_O_4_. After Li deposition, fitting reveals a significant change of the ratio of Co^3+^ : Co^2+^ to 1 : 1.4. The full lithiation of the cobalt oxide from Co^2+^Co^3+^_2_O^2−^_4_ to Li^+^Co^2+^_2_Co^3+^O^2−^_4_ would result in an inversion of the relative amount of Co^2+^ to Co^3+^ species, hence from the experimental results we assume at least a partial lithiation of the cobalt oxide. However, based on the Co 2p spectra we cannot resolve wether this modification is the result of an insertion reaction of Li into Co_3_O_4_ to form Li_n_Co_3_O_4_, of Li adsorption, a partial transformation of Co_3_O_4_ to CoO or a combination thereof.

**Figure 7:**
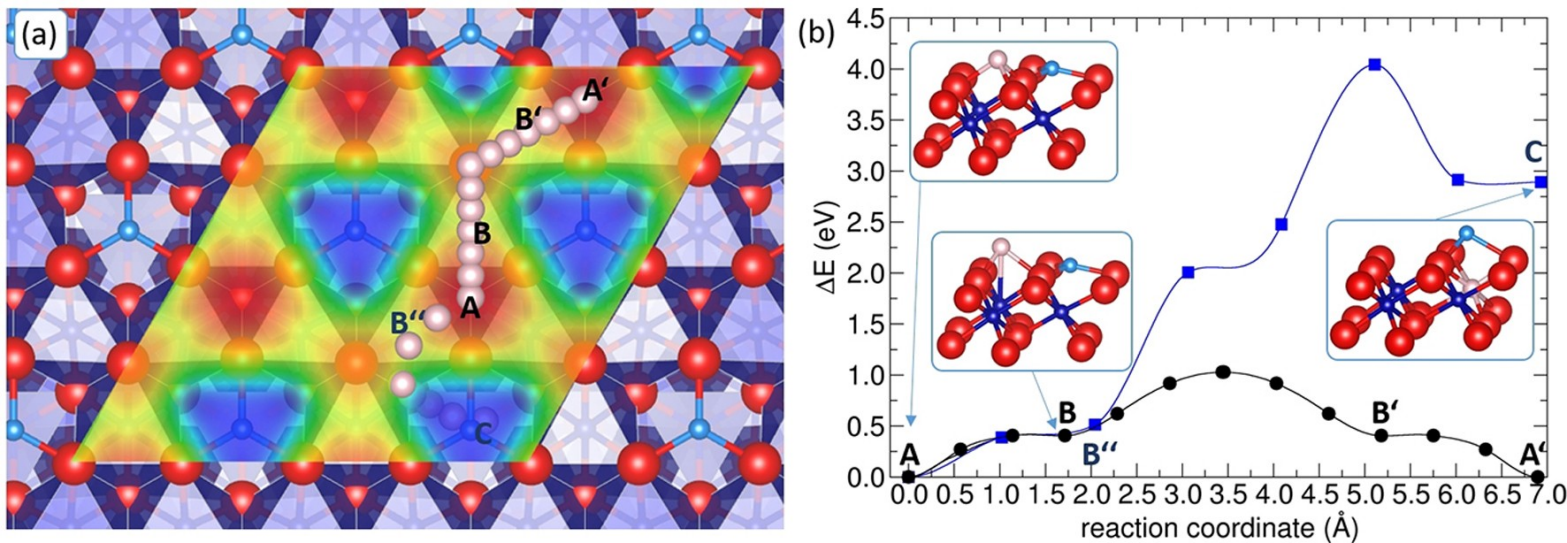
Top view onto the diffusion path of Li on Co_3_O_4_(111) (A−B−B′‐A′) and into Co_3_O_4_(111) (A−B′′‐C) (a) and the corresponding minimum energy paths (b). In (a) the electrostatic potential mapped onto an isosurface of the charge density (isosurface value=0.002 e/Å^3^) of the bare Co_3_O_4_(111) surface is inserted. The insets in (b) show sections of structures of the Li adsorption at the most stable adsorption site A, of the local minimum at adsorption site B and of Li inserted into a vacant octahedral site underneath the topmost oxygen layer (C).

DFT‐D3 calculations of the adsorption (or insertion) of Li atoms at (or in) Co_3_O_4_(111) allow us to gain further insights into the processes at the interface (Figure 7). Assuming mainly ionic interactions between Li and Co_3_O_4_(111) in the analysis, the stable adsorption sites can be anticipated by looking at the electrostatic potential of the bare surface: the electrostatic potential (of the electron) mapped onto an isosurface of the charge density is shown in Figure 7a: it is most positive (i. e. it denotes the region where a positive test charge preferably adsorbs) at the threefold hollow site of the O atoms adjacent to the surface terminating Co^2+^ atoms (red region, site A in Figure 7a). Indeed, this is the most stable adsorption site of Li at coverage of 0.9 Li atoms/nm^2^. The distance to the nearest O‐atoms is 1.9 Å, comparable to the Li−O distance in lithia (2.0 Å). The adsorption energy per Li atom is given relative to the energy of the bare surface and bulk Li metal: it amounts −3.47 eV (or −3.64 eV, if no dispersion corrections are employed for the bulk metal) at a coverage of 0.9 Li atoms/nm^2^ and decreases to −3.01 eV (or −3.18 eV, if no dispersion corrections are employed for the bulk metal) if all adsorption sites A are occupied (i. e. at a coverage of 3.5 Li atoms/nm^2^). This clearly reveals the anticipated strongly ionic character of the bond of Li to the substrate. Finally, the ionic character is shown by the calculation of the change of the atomic charges (determined by a Bader charge analysis) due to Li adsorption at a coverage of 3.5 Li atoms/nm^2^. It shows that charge is transferred from Li atoms (Δq/atom=+0.9 e) to the topmost Co^2+^ atoms (Δq/atom=−0.11 e) and O atoms (Δq/atom=−0.16 e), but also to directly underneath located octahedrally coordinated Co atoms (Co^3+^) (Δq/atom=−0.04 e). The atomic charge of the latter atoms reduces from 1.33 e to 1.29 e, which corresponds to the value of Co^2+^ atoms in the bulk (see section 3.1).

Furthermore, Li diffusion between the most stable adsorption sites A has been calculated at a Li coverage of 0.9 Li atoms/nm^2^. The diffusion path passes a shallow local minimum or saddle point (site B in Figure 7) which is 0.40 eV higher in energy than the most stable adsorption site. The barrier for the diffusion from site A to site B is 0.41 eV and thus only slightly higher than the energetic difference between the sites A and B. At site B Li is again located in a hollow position of the oxygen sublattice. Yet, the underlying octahedral site is occupied by Co. The distance to this Co atom only amounts to 2.28 Å. Further diffusion to the symmetry equivalent position B occurs via a top position of the oxygen sublattice. This configuration represents the transition state for Li diffusion on Co_3_O_4_(111). It is associated with a barrier of 1.0 eV which is higher than the value that was reported for Li diffusion along octahedral sites in the Co_3_O_4_ bulk by PBE calculations (0.75 eV).^[27]^ It is also higher than the value of 0.74 eV that has been reported for Pb diffusion on Co_3_O_4_(111).^[80]^ Surface structures in which Li is inserted into the Co_3_O_4_(111) surface slab have also been studied. The most stable surface structure in which Li is underneath the topmost O atoms is about 2.9 eV less stable than the adsorption of Li (see Figure 7b, structure C). In this insertion structure, Li occupies a vacant octahedral site. Li insertion into sub‐surface tetrahedral sites, which need to be passed by the Li atom to get to the octahedral sites, does not lead to any local minimum. Accordingly, a large barrier of 4.0 eV is obtained for the diffusion from adsorption site A to the most stable sub‐surface site C.

Considering the fact that the diffusion barrier at the surface is rather high and diffusion into the bulk through the defect free (111) surface is difficult, we conclude that at least a small part of the Co^3+^ reduction seen in XPS experiments must be due Li adsorption. Yet, the amount of reduced Co atoms solely due to Li adsorption would explain the experimentally found ratio of Co^3+^ : Co^2+^ of 1 : 1.4 only up to a depth of about 10 Å into Co_3_O_4_. Further contribution most likely stems from insertion reactions of Li into Co_3_O_4_ via step edges or defects (see flat island structure of Co_3_O_4_(111) in Figure 3).

Different from the Li deposition on CoO(111) (pristine and IL pre‐covered),^[26]^ we do not find any indication for the formation of metallic Co^0^ (∼778 eV). Here, we tentatively assume that surface reaction products (SEI) and maybe inserted (adsorbed) Li in Co_3_O_4_ in the surface region (on the surface) and in particular at the step edges inhibit further Li diffusion into the bulk, or the diffusion is at least very slow.

We finally compare with previous results on oxidic model electrodes measured in our laboratory, i. e., the interaction of a pre‐adsorbed BMP‐TFSI monolayers with post‐deposited Li on TiO(110),^[32]^ Li_4_Ti_5_O_12_(111)^[81]^ and CoO(111).^[26]^ First of all, on all investigated model electrodes, contact of an adsorbed IL with Li instantaneously results in BMP‐TFSI decomposition (cf. ref. [14]) and second in changes of the chemical state of the model electrode, i. e., Ti^4+^ partially transforms into Ti^3+^ species for TiO(110) and Li_4_Ti_5_O_12_(111), while Co^2+^ transforms into Co^0^. Also in the latter studies, the IL adayer/decomposition products are at least in parts permeable for Li, allowing for the reaction with TiO_2_(110), Li_4_Ti_5_O_12_(111) and CoO(111) surfaces. Differently from CoO(111), where Co^2+^ is transformed to Co^0^, the reduction of Ti^4+^ proceeds only to the Ti^3+^ stage. In the latter cases, reaction of Li with the substrate was found to take place both in the absence and the presence of an adsorbed IL adlayer,[^26^, ^32^] however, in the presence of an adsorbed IL adlayer, higher amounts of Li seem to be needed to observe a modification of the chemical state of the substrate,^[26]^ which is a hint that the IL adlayer/decomposition products could inhibit Li diffusion towards the substrate.

Overall, the measurements in this work reveal that stepwise postdeposition of Li on a Co_3_O_4_(111) thin film on Ir(100) covered with a pre‐adsorbed BMP‐TFSI adlayer (1.5–2 ML) at r.t. results in the gradual decomposition of BMP‐TFSI, which can be considered as the initial stage of the chemical SEI formation at the EEI in the absence of an electric field, i. e., at the open circuit potential (OCP). Both the anions and cations gradually decompose upon stepwise increasing the Li exposure. The efficient SEI formation at r.t. reflects a rather low barrier for IL decomposition upon interaction with Li. Simultaneously with IL decomposition (SEI formation) the chemical state of Co_3_O_4_(111) is modified in the near surface region, which we either assign to the insertion of Li into Co_3_O_4_ leading to Li_n_Co_3_O_4_ structures (0<n≤3, see ref. [27,28]), which partially show localized CoO−Li_2_O character,^[28]^ or a phase transformation into CoO or a combination thereof. Higher Li exposures do not induce significant changes of the chemical composition of the SEI and the chemical state of the thin film model electrode in the extended surface region (ID of ∼6 to 9 nm). Different from the Li deposition on CoO(111) (pristine and IL pre‐covered),^[26]^ we did not find the formation of metallic Co^0^. Thus, the conversion reaction is most likely inhibited (or diffusion is very slow) by IL/decomposition products (SEI) and/or inserted/adsorbed Li particularly at the steps. These experiments thus reproduce not only the initial stages of SEI formation (surface chemistry) but also the first lithiation step of Co_3_O_4_ under idealized conditions, much better defined than the complex situation in a battery cell.

## 3 Conclusion

In order to get deeper insights into the processes at the model electrode electrolyte interface in LIBs, we have investigated the interaction of ultrathin films of the ionic liquid BMP‐TFSI (model solvent/electrolyte) with Li (effect of the Li^+^ shuttle) on a Co_3_O_4_(111) thin film model anode and also the changes of the chemical state of Co_3_O_4_(111). Our main results and conclusions are:

1. RPBE‐D3+U is a suitable method to describe both the interactions within BMP‐TFSI and Co_3_O_4_.
2. XPS and STM experiments prove the formation of Co_3_O_4_(111) thin films with extended flat island structures.
3. Based on the XP spectra BMP‐TFSI adsorbs as molecular species on Co_3_O_4_(111) at r.t., except of a small amount of decomposition products which we tentatively assign to the interaction with the steps.
4. Stepwise postdeposition of small amounts of Li on a Co_3_O_4_(111) surface pre‐covered with a BMP‐TFSI adlayer instantaneously results in TFSI and BMP decomposition for the initial Li doses, reflecting the rather low reaction barrier for IL decomposition upon interaction with metallic Li. This process is considered as the initial stage of the chemical SEI formation at the EEI. XPS measurements reveal possible IL decomposition products of the TFSI anion such as LiCN, Li_2_NSO_2_CF_3_, LiF, Li_2_S, Li_2_O_2_, Li_2_O, etc. The decrease of the total peak area of adsorbate‐related peaks during Li deposition indicates that beside decomposition also desorption takes place.
5. The DFT‐D3+U calculations reveal that the decomposition of TFSI is more favorable than the reaction of BMP with Li. Li_2_NSO_2_CF_3_ and in particular LiCN are two of the most stable initial decomposition products. Decomposition products of BMP are less stable; thermodynamically, the formation of Li clusters is at least as favorable. Note, however, that due to a low barrier for the reaction of Li with BMP‐TFSI kinetic products such as Li_2_NCH_3_C_4_H_9_^+^ or LiNCH_3_C_4_H_9_ are possible, which can form by ethylene molecules desorbing into the gas phase in agreement with the observation of desorption in the XPS measurements.
6. After initial post‐deposition of Li to a pre‐adsorbed BMP‐TFSI adlayer, the XP Co 2p core level spectra show the formation of an increasing amount of Co^2+^ states relative to Co^3+^, which is attributed to charge transfer from Li^0^ to Co^3+^. This could be due to a partial lithiation of the cobalt oxide to Li_n_Co_3_O_4_ and/or Li adsorption. Furthermore, we cannot exclude that also a partial transformation of Co_3_O_4_ to CoO takes place. Larger Li deposits do not induce further significant changes in the Co 2p range, most likely because surface reaction products and maybe inserted/adsorbed Li in Co_3_O_4_ in the surface region, in particular at the step edges may either inhibit further Li diffusion into the bulk or the diffusion is at least very slow. The formation of Co^0^ due to a conversion reaction of CoO to Co^0^ and Li_2_O could not be observed in the present experiments.
7. DFT‐D3+U calculations reveal that the diffusion barrier at the Co_3_O_4_(111) surface is rather high and diffusion into the bulk through the defect free (111) surface is due to a prohibitively large barrier very unlikely. Hence, we assume that at least a small part of the Co^3+^ reduction seen in XPS experiments must be due to Li adsorption. Further contributions most likely stem from insertion reactions of Li into Co_3_O_4_ via step edges or defects.

In summary, the present surface chemistry model study reveals molecular details of the interactions of Li at the Co_3_O_4_(111) surface covered with an ultrathin IL layer. It provides valuable information about the basic processes occurring at the EEI which might be crucial for the further development of future batteries.

## Conflict of interest

The authors declare no conflict of interest.

## Supporting information

As a service to our authors and readers, this journal provides supporting information supplied by the authors. Such materials are peer reviewed and may be re‐organized for online delivery, but are not copy‐edited or typeset. Technical support issues arising from supporting information (other than missing files) should be addressed to the authors.

Supplementary
