# Supplementary material for: Model Studies on the Formation of the Solid Electrolyte Interphase: Reaction of Li with Ultrathin Adsorbed Ionic‐Liquid Films and Co3O4(111) Thin Films
Source: Chemphyschem. 2021 Feb 10;22(5):441–54. doi: 10.1002/cphc.202001033 (PMC7986933; doi:10.1002/cphc.202001033)
Supplement: Supplementary file 1 — Supplementary [file CPHC-22-441-s001.pdf]

ChemPhysChem

Supporting Information

Model Studies on the Formation of the Solid Electrolyte Interphase: Reaction of Li with Ultrathin Adsorbed Ionic-Liquid Films and Co_3O_4(111) Thin Films

Katrin Forster-Tonigold[+], Jihyun Kim, Joachim Bansmann, Axel Groß, and Florian Buchner[+]*

Supporting Information

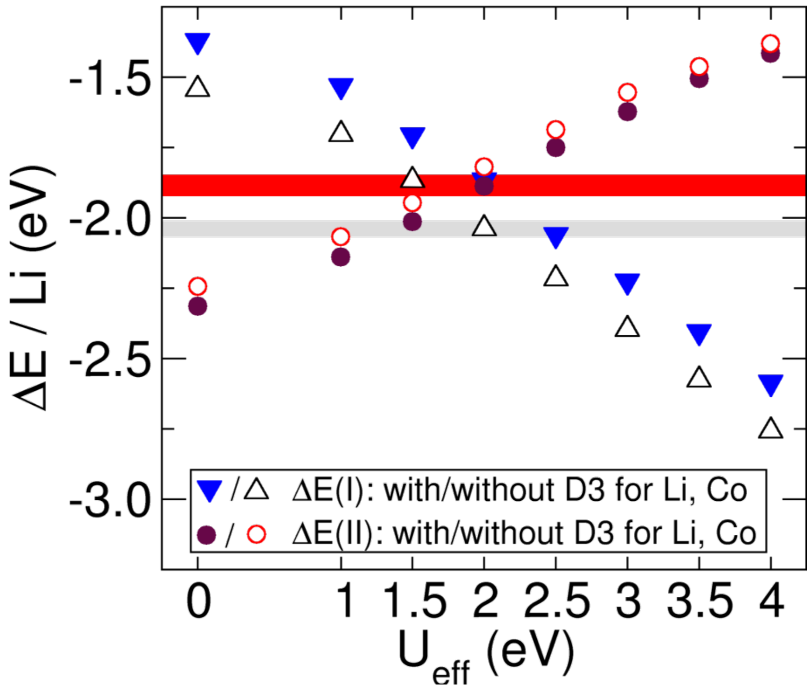

Figure S1. The reaction energy per Li atom ΔE/Li of the conversion reactions Co_3O_4 + 2Li → Li_2O + 3 CoO (I) and CoO + 2Li → Co + Li_2O (II) are shown as a function of the U_{eff} value. Horizontal lines or ranges denote experimentally determined values (grey: ΔE(I), red: ΔE(II)). ΔE/Li is calculated by employing the dispersion correction either to all compounds (filled symbols) or only to non-metallic compounds of the reaction (open symbols).

The screening of electrodynamic dispersion interactions in metals is not well described in the semiempirical dispersion correction scheme, leading to an overestimation of the interactions in metals that are already well described by pure GGA functionals. Therefore, the reaction energies ΔE(I,II) are also calculated with respect to the energies of the corresponding bulk metals (E(Li), E(Co)) that have been derived by RPBE+U calculations without dispersion corrections (open symbols in Figure S1). Then, U_{eff}=2.0 eV yields energies for reactions (I) and (II) that agree both very well with reaction energies that have been calculated based on the experimentally deduced enthalpies of formation of the compounds.

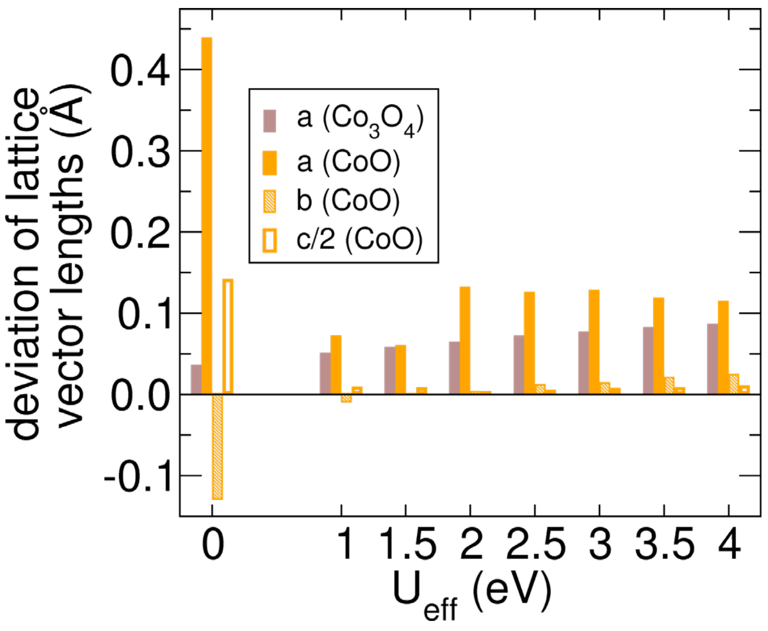

Figure S2. The deviation of the lattice parameters of Co_3O_4 and CoO from their experimentally determined values (Co_3O_4: 8.08 Å, CoO: a=5.182 Å, b=3.018 Å, c=3.019 Å, b=125.58°)[1-2] is shown as a function of the U_{eff} value.

When looking at the geometry of the cobalt oxides (see Figure S2) the largest discrepancy of the lattice constants with regard to the experimental values[1] is found for CoO when the pure RPBE-D3 method is used. The experimental lattice vectors a=5.182 Å and c/2=3.019 Å are overestimated by 8% and 5%, respectively, b=3.018 Å is underestimated by 4%. By adding the Hubbard like term U the deviation decreases to at most 2% for lattice vector a at U_{eff}=2.0 eV. The experimental lattice vector of Co_3O_4 (a=8.08 Å)[2] is overestimated by 0.4% using the pure RPBE-D3 method and the deviation increases almost linearly with an increasing value of U_{eff}. For U_{eff}=4 eV the lattice vector is still only overestimated by 1%.

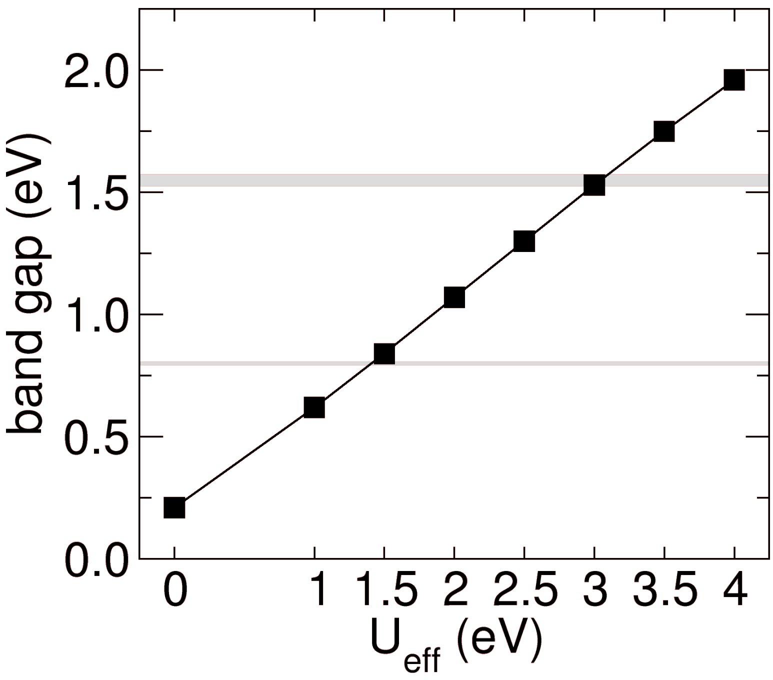

Figure S3. The band gap of Co_3O_4 is shown as a function of the U_{eff} value. The horizontal grey lines or ranges denote experimentally determined values according to Ref.[3-4] (0.76-0.82 eV) or Ref.[5-6](1.5-1.6 eV).

Regarding the electronic structure of Co_3O_4 a band gap of about 0.8 eV has been reported by calculations using both a hybrid functional and a many-body Green's function approach.[7] Values of 0.76 eV and 0.82 eV for the band gap have also been found in experimental studies.[3-4] Using this as a reference, a value of U_{eff}=1.5 eV for the Hubbard-like term appears to be a good choice to describe the electronic properties of Co_3O_4 (see Figure S3). However, the experimentally determined value of the band gap is not unambiguous; for example, values of 1.50-1.60 eV[5-6] have been reported as well.

Reference List

[1] W. Jauch, M. Reehuis, H. J. Bleif, F. Kubanek, P. Pattison, *Phys. Rev. B* **2001**, *64*, 052102.

[2] X. Liu, C. T. Prewitt, *Phys. Chem. Minerals* **1990**, *17*, 168-172.

[3] L. Qiao, H. Y. Xiao, H. M. Meyer, J. N. Sun, C. M. Rouleau, A. A. Puretzky, D. B. Geohegan, I. N. Ivanov, M. Yoon, W. J. Weber, M. D. Biegalski, *J. Mater. Chem. C* **2013**, *1*, 4628-4633.

[4] M. M. Waegele, H. Q. Doan, T. Cuk, *J. Phys. Chem. C* **2014**, *118*, 3426-3432.

[5] C.-S. Cheng, M. Serizawa, H. Sakata, T. Hirayama, *Mater. Chem. Phys.* **1998**, *53*, 225-230.

[6] K. J. Kim, Y. R. Park, *Solid State Commun.* **2003**, *127*, 25-28.

[7] V. Singh, M. Kosa, K. Majhi, D. T. Major, *J. Chem. Theory Comput.* **2015**, *11*, 64-72.
